# Micronutrient Supplementation and Clinical Outcomes in Patients with Dengue Fever

**DOI:** 10.4269/ajtmh.20-0731

**Published:** 2020-11-30

**Authors:** Steven D. Langerman, Mija Ververs

**Affiliations:** Centers for Disease Control and Prevention, Atlanta, Georgia

## Abstract

Dengue fever (DF) is a viral infection that is common in tropical countries and represents a significant cause of global morbidity and mortality. Despite its prevalence and severity, treatment options for DF remain limited and consist primarily of supportive measures. Several recent studies have concluded that micronutrient supplementation may improve clinical outcomes in patients with DF, but no review has summarized and synthesized these findings. We conducted a literature review to identify articles investigating the effect of micronutrient supplementation on clinical outcomes among patients with DF. We found several studies which indicated that supplemental vitamin C, vitamin D, vitamin E, and zinc may be useful adjuncts in DF treatment. Folic acid supplementation did not appear to affect clinical outcomes. The reviewed studies have significant limitations including small sample sizes and limited data about the baseline nutritional status of study subjects. We identify a need for additional high-quality randomized trials to elucidate the role of micronutrient supplementation in DF treatment.

## INTRODUCTION

Dengue fever (DF) is a mosquito-borne viral infection that is common in tropical and subtropical areas worldwide.^1^ Symptoms are variable but often include fever, rash, retro-orbital pain, thrombocytopenia, and severe myalgias.^2^ Although DF is often self-limited, some DF patients will progress to dengue hemorrhagic fever, a syndrome characterized by plasma leakage, changes in consciousness, and internal bleeding. Dengue hemorrhagic fever can ultimately lead to dengue shock syndrome, a severe condition consisting of circulatory collapse and sometimes death.^3^ The pathophysiology of these syndromes is complex, but dengue hemorrhagic fever and dengue shock syndrome are thought to be caused in part by an excessive cytokine response leading to increased vascular permeability, which contributes to vascular leakage and subsequent shock.^4^ Growing evidence also suggests that oxidative stress may play a role in DF pathogenesis.^5^

Dengue fever and its complications are a significant cause of global morbidity and mortality, with approximately 300 million infections and 10,000 deaths per year.^6–9^ Treatment options for DF patients remain limited, as no effective antiviral drugs are currently available.^10^ Current guidelines for clinical treatment of DF patients largely focus on supportive measures, with specific attention paid to management of fluid status and hemodynamic parameters among those patients who develop dengue hemorrhagic fever and dengue shock syndrome.^11,12^

Providers caring for DF patients sometimes provide micronutrient supplements as an adjunct to standard supportive care,^13–15^ as these supplements are generally inexpensive and some may offer hypothetical benefits to dengue patients via numerous potential mechanisms, including modulation of the host immune response^16–20^ and antioxidant effects.^13,21^ Proposed mechanisms of clinical benefit in DF for several micronutrients are outlined in Table 1.

**Table 1:** Micronutrients and proposed mechanisms of clinical benefit in patients with dengue fever

Micronutrient	Proposed mechanisms of clinical benefit
Vitamin A	Facilitates B-cell proliferation and T-cell activation; affects the activity of macrophages and natural killer cells^15^
Vitamin C	Scavenging of reactive oxygen species, increase in interferon production, facilitation of leukocyte phagocytic functions^13^
Vitamin D	Reduction of destructive inflammatory reactions via modulation of toll-like receptors (TLR3 and TLR9), increased production of Interleukin-10, increased expression of suppressor of cytokine signaling proteins^16^
Vitamin E	Protection of cell membranes from oxidative damage, scavenging of peroxyl radicals, enhancement of immune function via enzyme activation and changes in gene expression^21^
Folic acid	Hematinic effects, promotion of hematologic recovery^14^
Zinc	Facilitation of lymphocyte maturation and cytokine production, promotion of T-cell and neutrophil activity, acceleration of apoptosis^15^

Several recent studies have sought to evaluate the efficacy of micronutrient supplementation among DF patients. These studies have investigated associations between clinical outcomes and supplementation of various micronutrients, including vitamin C,^13,22^ vitamin D,^23^ vitamin E,^21,24^ folic acid,^14^ and zinc.^25^ No prior literature review has summarized the findings of these clinical studies. Our goals in this review, therefore, were to consolidate and synthesize the findings of studies between 2010 and 2020, which investigated the role of micronutrient supplementation in the clinical care of patients with DF, and to identify gaps in the current literature to guide future research on this topic.

## METHODS

We conducted a literature search using five major databases: MEDLINE, Global Health, Scopus, Academic Search Complete, and Embase. Search terms including “dengue” and “breakbone fever” were paired with micronutrient-related terms such as “vitamin” and “supplement” and the names of specific micronutrients, including “zinc,” “folate,” and “iron.” We restricted our search to human studies, English language articles, and articles published between January 1, 2010 and March 23, 2020. All abstracts and articles were managed through EndNote X9 (Clarivate Analytics, Philadelphia, PA). We initially de-duplicated records using the “Find Duplicates” feature in EndNote X9, and then screened each title for terms related to micronutrients and DF. We subsequently reviewed each abstract with possible relevance and excluded those in which micronutrient supplementation in human DF patients was not discussed. We then performed full-text review of each included article and removed those which did not contain novel findings relevant to our research question. Our complete search strategy is attached as Supplemental Appendix 1.

## RESULTS

**Figure 1.**
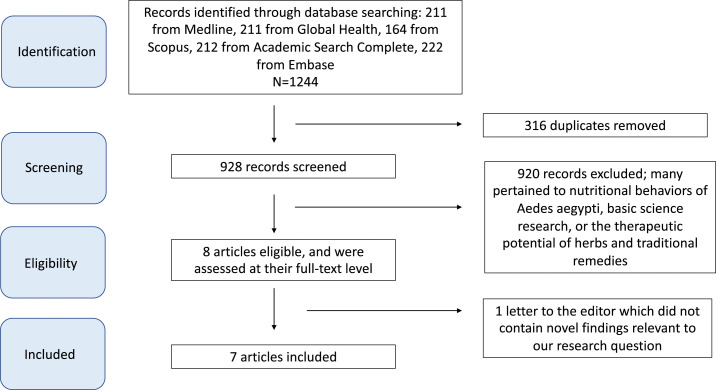
Flowchart summarizing literature search and selection process for review of micronutrient supplementation and clinical outcomes in patients with dengue fever (see separate file). This figure appears in color at www.ajtmh.org.

We identified a total of 1,244 records (Figure 1); 928 remained after de-duplication. We excluded 920 after screening for abstracts referencing associations between micronutrient supplementation and clinical outcomes among patients with DF. Content that caused exclusion included studies on the nutritional patterns of *Aedes aegypti* mosquitoes, articles commenting on the use of traditional herbal remedies to manage DF, and research investigating the impact of micronutrients on dengue-infected cells in vitro. We completed full-text review of the remaining eight records. One additional record was then excluded, as it was a letter to the editor that did not present any novel findings relevant to our research question. The remaining seven studies (six original articles and one conference abstract) were included in our review (Table 2). They included two observational studies investigating vitamin C, one randomized controlled trial (RCT) investigating vitamin D, one RCT and one prospective randomized open blinded evaluation of vitamin E, one observational study investigating folic acid, and one RCT investigating zinc.

**Table 2:** Description of studies evaluating micronutrient supplementation in DF patients

Micronutrient studied	Year	Author	Publication type	Title	Objective	Study type	Global rating of study quality*	Study location	Description of study site	Demographics of study subjects	Sample size	Daily dose	Duration of administration	Route of administration	Nutritional status of study subjects	Assessment of micronutrient deficiency at baseline?	Side effects of Drug	Study findings
Vitamin C	2019	Ramalingam	Journal article	A retrospective study on the effect of Vitamin C in the management of DF in three different states of India	To analyze the effect of vitamin C in the management of DF in the tertiary care hospitals of three selected states of India	Retrospective, observational	Weak	India	Various hospitals	35.5% of patients aged 0–15 years. 58.5% male; 41.5% female. No other demographic information included	200 (100 received vitamin C and standard care; 100 received standard care)	Not stated	Not stated	Variable (67 oral, 28 intravenous, 5 both)	No information provided	No	Not stated	Patients receiving vitamin C had 1) a shorter duration of hospital stay than those who did not receive vitamin C (*P* = 0.00), and 2) a greater percentage increase in platelet count than those who did not receive vitamin C (363.12% vs. 105.70%; *P* = 0.00)
Vitamin C	2018	Rammohan	Conference abstract	The efficacy of vitamin C on hemoglobin levels and WBC count as an adjuvant in the treatment of DF	To observe any effects of vitamin C on hemoglobin percentage and WBC count in patients with DF	Prospective, observational	N/A	Bangalore, Karnataka, India	Urban academic medical center (Rajarajeswari Hospital)	No demographic information included	123 (63 received vitamin C and standard care; 60 received standard care)	2000 mg (500 mg, four times daily)	Not stated	Oral	No information provided	No	Not stated	Patients receiving vitamin C had a significantly greater increase in WBC count (*P* = 0.00) than those who did not receive vitamin C
Vitamin D	2017	Zaman	Journal article	Effectiveness of vitamin D in the prevention of dengue haemorrhagic fever and dengue shock syndrome	To compare the risk and severity of development of DHF and dengue shock syndrome in patients receiving vitamin D supplement compared with those not receiving it	Randomized controlled trial	Weak	Rawalpindi, Punjab, Pakistan	Urban academic medical center (Benazir Bhutto Hospital)	Mean age: 33.43 ± 16.20 years. No other demographic information included	124 (62 received vitamin D and standard care, 62 received standard care)	200,000 IU once daily	Once	Not stated	Mean albumin at admission (g/L): 4.44 ± 3.65. Mean calcium at admission (mg/dL): 8.81 ± 0.42	No	Not stated	Those not receiving vitamin D were more likely to develop dengue hemorrhagic fever: χ^2^ = 16.43, *P* = 0.00. The relative risk of progressing to dengue hemorrhagic fever in patients who received vitamin D compared to those who did not receive vitamin D was 0.0588 (95% CI: 0.0081, 0.4285)
Vitamin E	2017	Chathurangana	Journal article	Effects of vitamin E supplementation on the clinical outcome of DF and dengue haemorrhagic fever in children	To evaluate the effects of vitamin E supplementation on the clinical course of DF and DHF in 5- to 12-year-old Sri Lankan children	Randomized controlled trial	Moderate	Colombo, Sri Lanka	Tertiary care hospital	Intervention group: mean age = 8.39 ± 2.16 years; control group: mean age = 8.35 ± 2.03 years	127 (61 received vitamin E and standard care; 66 received a placebo and standard care)	200 mg once daily (children 5–9 years of age); 400 mg once daily (children 10–12 years of age)	Seven days	Not stated	No information provided	No	No significant side effects were reported in treatment or placebo group	Treatment group had shorter mean duration of capillary leaking (33.5 hours vs. 44.8 hours; *P* = 0.02), a higher mean WBC count on day 6 (6.453 vs. 5.100; *P* = 0.02), a higher mean albumin on day 2.5 (45.8 vs. 41.5; *P* = 0.00) day 5, a lower mean packed cell volume at days 3 (38.36 vs. 39.66; *P* = 0.01) and 3.5 (38.80 vs. 39.93; *P* = 0.00), a lower mean alanine transaminase level on days 4 (40.8 vs. 92.0; *P* = 0.01) and 5 (54.8 vs. 101.2; *P* = 0.03), and a lower mean aspartate transaminase level on days 3 (67.7 vs. 110.5; *P* = 0.02), 4 (82.1 vs. 117.7; *P* = 0.01), and 5 (91.8 vs. 182.5; *P* = 0.00). No difference in 1) duration of hospital stay, or 2) occurrence of capillary leakage
Vitamin E	2012	Vaish	Journal article	Effect of vitamin E on thrombocytopenia in DF	To evaluate the effect of vitamin E on thrombocytopenia in DF	Prospective randomized open-blinded evaluation	Moderate	Lucknow, Uttar Pradesh, India	Tertiary teaching hospital	Intervention group: mean age = 28.65 ± 12.33 years. Age ranges: < 20 years (8); 20–40 years (14); 40–60 years (6); > 60 years (2). Male:female ratio 7.5:1. Control group: mean age = 31.67 ± 11.34 years. Age ranges: < 20 years (7); 20–40 years (16); 40–60 years (6); > 60 years (1). Male:female ratio 5:1	59 (31 received vitamin E and standard care, 28 received standard care)	400 mg once daily	Not stated	Not stated	No information provided	No	Not stated	On days 4 and 7, the mean platelet counts in the group receiving vitamin E were 122.19 × 103/mm^3^ and 217.35 × 103, respectively, which were significantly higher than the mean platelet counts on those days in the control group (92.57 × 103/mm^3^ and 146.78 × 103/mm^3^, respectively). *P* (day 4) = 0.04 and *P* (day 7) = 0.00
Folic acid	2019	Syed	Journal article	The use of folic acid in dengue: Has it any value?	To compare the duration of recovery of thrombocytopenia in patients with dengue infection who received folic acid and those who did not	Retrospective, observational	Weak	Karachi, Pakistan	Urban academic medical center (Aga khan university hospital)	All enrolled patients were aged > 16 years. Mean age 36.5 ± 15.0 years. No other demographic information included	1,464 (1,322 received folic acid and standard care; 142 received standard care)	5 mg once daily	Not stated	Not stated	No information provided	No	Not stated	No significant differences in length of hospitalization, creatinine levels, the time it took platelets to double their nadir, likelihood of needing admission to the intensive care unit, or mortality between the experimental group and the control group
Zinc	2018	Rerksuppaphol	Journal article	A randomized controlled trial of zinc supplementation as adjuvant therapy for dengue Viral infection in Thai children	To assess the effects of zinc supplementation on dengue virus infection outcomes	Randomized controlled trial	Strong	Bangkok, Thailand	Urban academic medical center (Srinakharinwirot University Hospital)	Mean age 6.3 years (range: 1.1–13.8 years). 62% male; 38% female. Treatment group: mean age: 6.6 ± 3.6 years; 56% male, 44% female. Control group: mean age: 5.9 ± 3.1 years; 68% male, 32% female	50 (25 received zinc and standard care; 25 received placebo and standard care)	45 mg (15 mg, three times daily)	Five days or until convalescence of fever	Oral	Treatment group: Mean BMI (kg/m^2^) = 16.07 (3.94); mean albumin (g/dL) 4.1; 48% of patients zinc deficient at baseline. Control group: Mean BMI (kg/m^2^) 16.30; mean albumin (g/dL) 4.0.	Yes. Treatment group: 48% of patient zinc deficient at baseline. Control group: 44% of patients zinc deficient at baseline. No statistically significant difference between the two groups	Yes. In treatment group, two patients reported nausea; one patient reported loose stool. In placebo group, two patients reported nausea	Shorter duration of hospital stay in group receiving zinc supplement compared with placebo group (62.5 hours vs. 84.7 hours, *P* = 0.01). No significant difference in mean time to defervescence between group receiving zinc supplement and placebo group

N/A = not applicable. * Effective Public Health Practice Project, 1998. Quality Assessment Tool for Quantitative Studies. Hamilton, ON: Effective Public Helath Practice Project. Available at: https://merst.ca/ephpp/.

BMI = body mass index; DF = dengue fever; DHF = dengue hemorrhagic fever; IU = international units; WBC = white blood cell.

Of the seven reviewed studies, six were assessed using the Quality Assessment Tool for Quantitative Studies (Effective Public Health Practice Project, Hamilton, ON, Canada).^26^ The seventh reviewed study was described only in a conference abstract, and thus the information necessary to evaluate it was not available. Of the six studies assessed, three received global ratings of “weak,” two received a global rating of “moderate,” and one received a global rating of “strong” (Supplemental Table 1). Study features that resulted in lower scores included failure to adequately address possible confounders, inadequate provision of information regarding withdrawals and dropouts, and lower-quality study types (e.g., observational studies).

### Vitamin C.

Ramalingam et al.^13^ and Rammohan et al.^22^ both investigated the effect of supplemental vitamin C in the management of DF. Ramalingam et al.^13^ conducted a retrospective study of 200 patients infected with dengue virus across various hospitals in India. The authors stated that 35.5% of patients were aged 0–15 years, but did not provide any other information regarding the ages of study participants. One hundred of these patients were given supplemental vitamin C and 100 were not, but information about dosing was not provided. Among the patients receiving vitamin C, 67% received oral vitamin C, 28% received intravenous (IV) vitamin C, and 5% (described by the authors as “more severe cases”) received both. The authors determined that the average increase in platelet count was greater among patients who received vitamin C than among those who did not (363% versus 105%; *P* = 0.00). Patients receiving vitamin C were also hospitalized significantly fewer days than those who did not receive vitamin C. Of patients who received vitamin C, 39% had hospitalizations lasting fewer than 5 days. Of those who did not receive vitamin C, only 3% had hospitalizations lasting fewer than 5 days. In describing outcomes, the authors did not differentiate between patients who received oral vitamin C and patients who received IV vitamin C. Of note, this study did not include any information about patients’ baseline nutritional status, did not state whether patients were deficient in vitamin C, nor did it contain any information about the patients’ diets.

Rammohan et al.^22^ conducted a prospective observational study of 123 DF patients with thrombocytopenia at an academic medical center in Bangalore, India. The results were summarized in a conference abstract, which did not provide effect sizes for the study’s results or any demographic information about the enrolled patients. Study subjects were randomized into two groups: patients in one group received standard care with no vitamin C (*n* = 60), whereas patients in the other group received standard care in addition to 500 mg of oral vitamin C four times/day (*n* = 63). The authors assessed hemoglobin and white blood cell (WBC) counts at day 7 of treatment. They found no change in hemoglobin in either group, and noted that WBC counts increased in both groups. White blood cell counts in the treatment group, however, increased significantly more than those in the nontreatment group (*P* = 0.00).

### Vitamin D.

Zaman et al.^23^ conducted an RCT of 124 patients suffering from DF at an academic medical center in Rawalpindi, Pakistan. The mean age of the study subjects was 33 years, but baseline data were not provided separately for the intervention and control groups. Patients were randomized either to receive a single dose of 200,000 international units of vitamin D (*n* = 62) in addition to standard care, or to receive standard care only (*n* = 62). Both groups were followed for development of dengue hemorrhagic fever or dengue shock syndrome. Among patients who received the vitamin D supplement, only one patient (1.6%) developed dengue hemorrhagic fever. In the group that did not receive the supplement, 17 patients (27%) developed dengue hemorrhagic fever. Those not receiving vitamin D were more likely to develop dengue hemorrhagic fever (χ^2^ = 16.43, *P* = 0.00). The relative risk of progressing to dengue hemorrhagic fever in patients who received vitamin D compared with those who did not receive vitamin D was 0.0588 (95% CI: 0.0081, 0.4285). However, this study did not assess whether patients were vitamin D deficient at baseline, and did not contain any information about the patients’ diets.

### Vitamin E.

Chathurangana et al.^21^ conducted an RCT of 127 children aged 5–12 years with DF at a tertiary care hospital in Colombo, Sri Lanka. They were randomized to one of two groups: 1) the experimental group, in which patients received either 200 mg of vitamin E once daily for 7 days (for children aged 5–9 years) or 400 mg of vitamin E once daily for 7 days (for children aged 10–12 years) in addition to supportive care; or 2) the control group, in which patients received only supportive care. A wide array of clinical, biochemical, and hematological parameters were measured across both groups including duration of capillary leaking, aspartate transaminase (AST), alanine transaminase (ALT), albumin, serum cholesterol, serum calcium, WBC count, platelet count, and packed cell volume (PCV). Each of these parameters was evaluated twice per day from day 2 to day 7 of hospitalization. The only statistically significant differences between the two groups were as follows: the group receiving vitamin E had a shorter mean duration of capillary leakage (33.5 hours versus 44.8 hours; *P* = 0.02), a higher mean WBC count on day 6 (6.453 versus 5.100; *P* = 0.02), a higher mean albumin on days 2.5 (45.8 versus 41.5; *P* = 0.00) and 5, a lower mean PCV at days 3 (38.36 versus 39.66; *P* = 0.01) and 3.5 (38.80 versus 39.93; *P* = 0.00), a lower mean ALT level on days 4 (40.8 versus 92.0; *P* = 0.01) and 5 (54.8 versus 101.2; *P* = 0.03), and a lower mean AST level on days 3 (67.7 versus 110.5; *P* = 0.02), 4 (82.1 versus 117.7; *P* = 0.01), and 5 (91.8 versus 182.5; *P* = 0.00). There was no difference in the 1) duration of hospital stay or 2) occurrence of capillary leakage. This study did not assess patients’ baseline nutritional status and did not evaluate for vitamin E deficiency at baseline.

Vaish et al.^24^ performed a prospective randomized open-blinded evaluation of 59 patients with DF at a tertiary teaching hospital in Lucknow, India. The intervention group included 31 DF patients who received 400 mg vitamin E once daily in addition to standard care, whereas the control group consisted of 28 patients who received standard care without vitamin E. Most study subjects were male, and the mean age of enrolled patients was 28.7 years in the intervention group and 31.7 years in the control group, with no statistically significant difference in mean age between the two groups. Platelet counts in both groups were measured on days 0, 1, 4, and 7 of illness. The authors found that mean platelet counts among those patients in the intervention group were significantly higher than those among patients in the control group on days 4 (122.19 × 10^3^ cells/mm^3^ versus 92.57 × 10^3^ cells/mm^3^; *P* = 0.04) and 7 (217.35 × 10^3^ cells/mm^3^ versus 146.78 × 10^3^ cells/mm^3^; *P* = 0.00) of treatment. Of note, this study did not provide information about patients’ baseline nutritional status, did not state whether they were vitamin E deficient at baseline, and did not assess whether patients experienced side effects.

### Folic acid.

Syed et al.^14^ conducted a retrospective observational study of 1,464 patients who were hospitalized with DF at an urban academic medical center in Karachi, Pakistan. Patients had a mean age of 36.5 years. The study’s primary goal was to determine whether there was a significant difference in the duration of recovery of thrombocytopenia between patients who received folic acid supplements and patients who did not. As secondary endpoints, they also investigated whether any differences existed in the mortality rate, likelihood of needing admission to the intensive care unit, length of stay, and creatinine levels between the two groups. Of the 1,464 patients, 1,322 (90.3%) received folic acid and 142 (9.7%) did not. There were no significant differences between the two groups with respect to any of the above endpoints, but the authors did not provide effect sizes. This study did not assess for baseline nutritional status, did not evaluate for zinc deficiency, and did not assess for side effects among patients receiving supplements.

### Zinc.

Rerksuppaphol and Rerksuppaphol^25^ conducted an RCT in 50 children (mean age 6.3 years) with DF at an academic medical center in Bangkok, Thailand. Patients were randomized to receive either: 1) 15 mg oral bis-glycinate zinc three times daily for 5 days or until defervescence, or 2) placebo. Baseline zinc deficiency was present in 48% of patients in the treatment group and 44% of patients in the control group, with no statistically significant difference between the two groups. The authors found no statistically significant difference in mean time to defervescence between the supplement group and the placebo group (29.2 hours versus 38.1 hour; *P* = 0.27). However, the mean duration of hospitalization was shorter in the supplementation group than in the placebo group (62.5 hours versus 84.7 hours; *P* = 0.01).

## DISCUSSION

In this review, we describe seven studies published between 2010 and 2020 which analyzed the association between micronutrient supplementation and clinical outcomes in patients with DF. We evaluated six of these studies using the Quality Assessment Tool for Quantitative Studies and found that they were of varying quality, with three receiving a global rating of “weak,” two receiving a global rating of “moderate,” and one receiving a global rating of “strong.” In all studies that provided dosing information, the doses used were between 2.63 times and 500 times higher than the daily recommended nutrient intake suggested by the WHO and the Food and Agriculture Organization of the United Nations.^27^ Vitamin C supplementation was associated with higher platelet counts,^13^ shorter duration of hospitalization,^13^ and greater increases in WBC counts.^22^ Vitamin D supplementation was associated with lower likelihood of progression to dengue hemorrhagic fever.^23^ Vitamin E supplementation was associated with improvements in various clinical and hematological parameters,^21^ including significantly higher subsequent platelet counts among patients admitted for thrombocytopenia.^24^ Folic acid supplementation was not associated with a change in clinical outcomes.^14^ Zinc supplementation was associated with shorter duration of hospitalization.^25^

There is limited previous research investigating relationships between micronutrient supplementation and clinical outcomes in DF patients. A 2014 review by Ahmed et al.^15^ summarized existing work on micronutrients and dengue, but at the time of its publication, there were only two articles evaluating the use of micronutrient supplements in the clinical treatment of DF patients. These two articles were the 2012 Vaish et al.^24^ study described in our review and a 2009 case series by Sánchez-Valdéz et al.^28^ The Sánchez-Valdéz et al. case series concluded that supplementation of vitamin D3 and calcium may result in “improved overall clinical condition and shorter duration of symptoms of DF.”^28^ This assessment, however, was based on only five cases and did not have a control group. The other articles included in the Ahmed et al.^15^ review described laboratory studies of dengue virus–infected cells,^29,30^ observational studies assessing associations between vitamin D receptor polymorphisms and DF disease in humans,^31,32^ laboratory studies of dengue virus–infected mice,^33–35^ and noninterventional studies analyzing the serum or plasma components of DF patients.^36–41^ None of these studies directly investigated any clinical interventions for DF. Our review, performed 6 years after the Ahmed et al.^15^ review was published, identified six new clinical studies assessing micronutrient supplementation among DF patients, which may reflect a growing interest in this topic.

Although six of the seven articles in this review identified clinical benefits of supplementation of vitamin C, vitamin D, vitamin E, and zinc in DF patients, these findings must be interpreted in light of the studies’ significant limitations. Of the seven studies described, five did not include any information about patients’ baseline nutritional status,^13,14,21,22,24^ and six did not state whether patients were deficient in the micronutrient to be supplemented.^13,14,21–24^ Five of the studies did not assess whether patients experienced any side effects related to the supplements.^13,14,22–24^ None of the seven studies contained any information about study subjects’ diets. Six studies had small sample sizes, with 200 or fewer total participants.^13,21–25^ One study did not provide any information about the micronutrient doses provided and pooled data from multiple hospitals using multiple routes of administration.^13^ One study investigated a wide array of laboratory parameters across many days and reported a handful of statistically significant results which may have limited clinical importance.^21^ Another study was described only in a conference abstract, which provided no effect sizes and contained very limited background information about the study population.^22^

In this review of the literature, we found studies demonstrating possible improvement of DF clinical outcomes associated with the supplementation of vitamin C, vitamin D, vitamin E, and zinc. However, these studies have significant limitations, including small sample sizes and failure to assess baseline nutritional status. As micronutrient supplementation may represent a simple, low-cost adjunct to standard medical care,^15^ there is a need for additional high-quality studies of this topic. To obtain the most robust evidence possible, we recommend RCTs with large sample sizes and clinically meaningful endpoints. Studies should provide clear baseline data regarding both intervention and control groups with respect to possible confounders. Rates of withdrawals and dropouts should be stated explicitly, and authors should provide effect sizes in their results. We further recommend that future studies use consistent micronutrient doses, provide detailed demographic data about study subjects, and clearly specify the frequency, duration, and route of administration for each supplement administered. In addition, it is essential that future research investigates whether the benefits of micronutrient supplementation are seen in all patients or only in those with specific nutritional deficiencies. Therefore, future studies should provide baseline data about study subjects’ nutritional status, such as relevant micronutrient deficiencies, anthropometrics, laboratory tests such as prealbumin, or inventories of nutritional intake. Information about the diet provided to hospitalized patients should also be included. Furthermore, we agree with the recommendations of Ahmed et al.^15^ that future studies should include both adults and children, and should assess the impact of nutritional interventions on dengue of all levels of severity using standardized WHO clinical severity case definitions. These steps will help to provide clearer evidence of the potential benefits micronutrient supplementation may offer to DF patients.

## Supplemental Appendix and table

Supplemental materials

## References

[1] Hosseini S Oliva-Ramirez J Vazquez-Villegas P Rodriguez-Garcia A Munoz-Soto RB Aghamohammadi N Martinez-Chapa SO, 2018 Dengue fever: a worldwide threat an overview of the infection process, environmental factors for a global outbreak, diagnostic platforms and vaccine developments. Curr Top Med Chem 18: 1531–1549. 30394209 10.2174/1568026618666181105130000

[2] Chaloemwong J Tantiworawit A Rattanathammethee T Hantrakool S Chai-Adisaksopha C Rattarittamrong E Norasetthada L, 2018 Useful clinical features and hematological parameters for the diagnosis of dengue infection in patients with acute febrile illness: a retrospective study. BMC Hematol 18: 20. 30181881 10.1186/s12878-018-0116-1 PMC6114047

[3] Ranjit S Kissoon N, 2011 Dengue hemorrhagic fever and shock syndromes. Pediatr Crit Care Med 12: 90–100. 20639791 10.1097/PCC.0b013e3181e911a7

[4] Malavige GN Ogg G, 2012 Pathogenesis of severe dengue infection. Ceylon Med J 57: 97–100. 23086023 10.4038/cmj.v57i3.4701

[5] Seet RC Lee CY Lim EC Quek AM Yeo LL Huang SH Halliwell B, 2009 Oxidative damage in dengue fever. Free Radic Biol Med 47: 375–380. 19427377 10.1016/j.freeradbiomed.2009.04.035

[6] Messina JP 2019 The current and future global distribution and population at risk of dengue. Nat Microbiol 4: 1508–1515. 31182801 10.1038/s41564-019-0476-8 PMC6784886

[7] Stanaway JD 2016 The global burden of dengue: an analysis from the Global Burden of Disease Study 2013. Lancet Infect Dis 16: 712–723. 26874619 10.1016/S1473-3099(16)00026-8 PMC5012511

[8] Messina JP Brady OJ Pigott DM Brownstein JS Hoen AG Hay SI, 2014 A global compendium of human dengue virus occurrence. Sci Data 1: 140004. 25977762 10.1038/sdata.2014.4 PMC4322574

[9] Murray NE Quam MB Wilder-Smith A, 2013 Epidemiology of dengue: past, present and future prospects. Clin Epidemiol 5: 299–309. 23990732 10.2147/CLEP.S34440 PMC3753061

[10] Nobori H Toba S Yoshida R Hall WW Orba Y Sawa H Sato A, 2018 Identification of compound-B, a novel anti-dengue virus agent targeting the non-structural protein 4A. Antivir Res 155: 60–66. 29758236 10.1016/j.antiviral.2018.05.003

[11] World Health Organization and UNICEF/UNDP/World Bank/WHO Special Programme for Research and Training in Tropical Diseases, 2012 Handbook for Clinical Management of Dengue. Geneva, Switzerland: WHO.

[12] Paterson DL Bonomo RA, 2005 Extended-spectrum beta-lactamases: a clinical update. Clin Microbiol Rev 18: 657–686. 16223952 10.1128/CMR.18.4.657-686.2005 PMC1265908

[13] Ramalingam K Varghese CS Elias C Mathew GM Balasubramanian A, 2019 A retrospective study on the effect of vitamin C in the management of dengue fever in three different states of India. Int J Res Pharm Sci 10: 2670–2673.

[14] Syed AA Zafar S Shah AA Safia A, 2019 The use of folic acid in dengue: has it any value? Trop Doctor 49: 85–87. 10.1177/0049475519827110 30755107

[15] Ahmed S Finkelstein JL Stewart AM Kenneth J Polhemus ME Endy TP Cardenas W Mehta S, 2014 Micronutrients and dengue. Am J Trop Med Hyg 91: 1049–1056. 25200269 10.4269/ajtmh.14-0142 PMC4228873

[16] Arboleda Alzate JF Rodenhuis-Zybert IA Hernandez JC Smit JM Urcuqui-Inchima S, 2017 Human macrophages differentiated in the presence of vitamin D3 restrict dengue virus infection and innate responses by downregulating mannose receptor expression. PLoS Negl Trop Dis 11: e0005904. 29020083 10.1371/journal.pntd.0005904 PMC5653353

[17] Arboleda JF Fernandez GJ Urcuqui-Inchima S, 2019 Vitamin D-mediated attenuation of miR-155 in human macrophages infected with dengue virus: implications for the cytokine response. Infect Genet Evol 69: 12–21. 30639520 10.1016/j.meegid.2018.12.033

[18] Jadhav NJ Gokhale S Seervi M Patil PS Alagarasu K, 2018 Immunomodulatory effect of 1, 25 dihydroxy vitamin D3 on the expression of RNA sensing pattern recognition receptor genes and cytokine response in dengue virus infected U937-DC-SIGN cells and THP-1 macrophages. Int Immunopharmacol 62: 237–243. 30032048 10.1016/j.intimp.2018.07.019

[19] Martinez-Moreno J Hernandez JC Urcuqui-Inchima S, 2020 Effect of high doses of vitamin D supplementation on dengue virus replication, toll-like receptor expression, and cytokine profiles on dendritic cells. Mol Cell Biochem 464: 169–180. 31758375 10.1007/s11010-019-03658-w

[20] Giraldo DM Cardona A Urcuqui-Inchima S, 2018 High-dose of vitamin D supplement is associated with reduced susceptibility of monocyte-derived macrophages to dengue virus infection and pro-inflammatory cytokine production: an exploratory study. Clin Chim Acta 478: 140–151. 29289621 10.1016/j.cca.2017.12.044

[21] Chathurangana PWP Samaranayake DBDL Quienters VG Wickramasinghe VP, 2017 Effects of vitamin E supplementation on the clinical outcome of dengue fever and dengue haemorrhagic fever in children. Asian Pac J Trop Dis 7: 645–649.

[22] Rammohan S Bhandare B Mv K, 2018 The efficacy of vitamin C on hemoglobin levels and white blood cell count as an adjuvant in the treatment of dengue fever. Conference: 3rd International Conference on Academic and Industrial Innovations: Transitions in Pharmaceutical, Medical and Biosciences. Goa, India: INNOPHARM3, Kala Academy.

[23] Zaman S Mahmud MR Khalid MA Zahid A Khalid S Kabir I Manzoor S Baqai HZ, 2017 Effectiveness of vitamin D in prevention of dengue haemorrhagic fever and dengue shock syndrome. J Rawalpindi Med Coll 21: 205–207.

[24] Vaish A Verma S Agarwal A Gupta L Gutch M, 2012 Effect of vitamin E on thrombocytopenia in dengue fever. Ann Trop Med Public Health 5: 282–285.

[25] Rerksuppaphol S Rerksuppaphol L, 2018 A randomized controlled trial of zinc supplementation as adjuvant therapy for dengue viral infection in Thai children. Int J Prev Med 9: 88. 30450171 10.4103/ijpvm.IJPVM_367_17 PMC6202777

[26] Effective Public Health Practice Project, 1998 Quality Assessment Tool for Quantitative Studies. Hamilton, ON: Effective Public Health Practice Project Available at: Https://merst.ca/ephpp/. Accessed June 2, 2020.

[27] World Health Organization, 2005 Vitamin and Mineral Requirements in Human Nutrition, 2nd edition Geneva, Switzerland: WHO.

[28] Sanchez-Valdez E Delgado-Aradillas M Torres-Martinez JA Torres-Benitez JM, 2009 Clinical response in patients with dengue fever to oral calcium plus vitamin D administration: study of 5 cases. Proc West Pharmacol Soc 52: 14–17. 22128411

[29] Puerta-Guardo H Medina F De la Cruz Hernandez SI Rosales VH Ludert JE del Angel RM, 2012 The 1alpha,25-dihydroxy-vitamin D3 reduces dengue virus infection in human myelomonocyte (U937) and hepatic (Huh-7) cell lines and cytokine production in the infected monocytes. Antivir Res 94: 57–61. 22387385 10.1016/j.antiviral.2012.02.006

[30] Shafee N AbuBakar S, 2002 Zinc accelerates dengue virus type 2-induced apoptosis in Vero cells. FEBS Lett 524: 20–24. 12135735 10.1016/s0014-5793(02)02991-5

[31] Alagarasu K Honap T Mulay AP Bachal RV Shah PS Cecilia D, 2012 Association of vitamin D receptor gene polymorphisms with clinical outcomes of dengue virus infection. Hum Immunol 73: 1194–1199. 22917542 10.1016/j.humimm.2012.08.007

[32] Loke H Bethell D Phuong CX Day N White N Farrar J Hill A, 2002 Susceptibility to dengue hemorrhagic fever in vietnam: evidence of an association with variation in the vitamin d receptor and Fc gamma receptor IIa genes. Am J Trop Med Hyg 67: 102–106. 12363051 10.4269/ajtmh.2002.67.102

[33] Shrivastava R Upreti RK Chaturvedi UC, 2005 Effects of dengue virus infection on the spleen of male mice given hexavalent chromium with drinking water. Toxicol Mech Methods 15: 323–329. 20021051 10.1080/153765291009732

[34] Shrivastava R Nagar R Ravishankar GA Upreti RK Chaturvedi UC, 2007 Effect of pretreatment with chromium picolinate on haematological parameters during dengue virus infection in mice. Indian J Med Res 126: 440–446. 18160748

[35] Shrivastava R Srivastava S Upreti RK Chaturvedi UC, 2005 Effects of dengue virus infection on peripheral blood cells of mice exposed to hexavalent chromium with drinking water. Indian J Med Res 122: 111–119. 16177467

[36] Alagarasu K Bachal RV Bhagat AB Shah PS Dayaraj C, 2012 Elevated levels of vitamin D and deficiency of mannose binding lectin in dengue hemorrhagic fever. Virol J 9: 86. 22559908 10.1186/1743-422X-9-86 PMC3413536

[37] Albuquerque LM Trugilho MR Chapeaurouge A Jurgilas PB Bozza PT Bozza FA Perales J Neves-Ferreira AG, 2009 Two-dimensional difference gel electrophoresis (DiGE) analysis of plasmas from dengue fever patients. J Proteome Res 8: 5431–5441. 19845402 10.1021/pr900236f

[38] Yuliana N Ryadi Fadil R Chairulfatah A, 2009 Serum zinc levels and clinical severity of dengue infection in children. Paediatr Indonesiana 49: 309–314.

[39] Widagdo, 2008 Blood zinc levels and clinical severity of dengue hemorrhagic fever in children. Southeast Asian J Trop Med Public Health 39: 610–616. 19058597

[40] Klassen P Biesalski HK Mazariegos M Solomons NW Furst P, 2004 Classic dengue fever affects levels of circulating antioxidants. Nutrition 20: 542–547. 15165617 10.1016/j.nut.2004.03.016

[41] Chaiyaratana W Chuansumrit A Atamasirikul K Tangnararatchakit K, 2008 Serum ferritin levels in children with dengue infection. Southeast Asian J Trop Med Public Health 39: 832–836. 19058577

