# Supplementary material for: Micronutrient Supplementation and Clinical Outcomes in Patients with Dengue Fever
Source: Am J Trop Med Hyg. 2020 Nov 30;104(1):45–51. doi: 10.4269/ajtmh.20-0731 (PMC7790074; doi:10.4269/ajtmh.20-0731)
Supplement: Supplementary file 1 [file tpmd200731.SD1.pdf]

Supplemental Table: Evaluation of Six Reviewed Studies Using the Quality Assessment Tool for Quantitative Studies[1]

Study	Selection Bias	Study Design	Confounders	Blinding	Data Collection	Withdrawals/ Drop-Outs	Global Rating
Ramalingam (2019)	Moderate	Weak	Weak	Moderate	Strong	Moderate	Weak
Zaman (2017)	Moderate	Strong	Weak	Weak	Strong	Weak	Weak
Chathurangana (2017)	Moderate	Strong	Strong	Moderate	Strong	Weak	Moderate
Vaish (2012)	Moderate	Strong	Weak	Moderate	Strong	Strong	Moderate
Syed (2019)	Moderate	Weak	Weak	Moderate	Strong	Moderate	Weak
Rerksuppahol (2018)	Strong	Strong	Strong	Moderate	Strong	Strong	Strong

1. Effective Public Health Practice Project, 1998. Quality Assessment Tool for Quantitative Studies. Hamilton, ON: Effective Public Health Practice Project. Available from: Https://merst.ca/ephpp/
