# Supplementary material for: Micronutrient Supplementation and Clinical Outcomes in Patients with Dengue Fever
Source: Am J Trop Med Hyg. 2020 Nov 30;104(1):45–51. doi: 10.4269/ajtmh.20-0731 (PMC7790074; doi:10.4269/ajtmh.20-0731)
Supplement: Supplementary file 2 [file tpmd200731.SD2.pdf]

Search performed by: Steven Langerman

Date of Search: 03/23/2020

Search Strategy:

Database	Strategy	Run Date	Records
Medline<br><br>(Ovid)<br><br>1946-	1. (dengue OR breakbone fever* OR break bone fever*).mp. OR exp Dengue/<br><br>2. (fortified OR micronutrient* OR supplement* OR vitamin* OR retinol OR retinoic acid* OR antioxidant* OR zinc OR iron OR thiamine OR riboflavin OR niacin OR pantothenic acid* OR pyridoxine OR biotin OR folic acid* OR folate OR cobalamin OR chromium OR magnesium).mp.<br><br>3. 1 AND 2<br><br>4. Limit 3 to animals<br><br>5. Limit 4 to humans<br><br>6. 4 NOT 5<br><br>7. 3 NOT 6<br><br>Limits: English, 2010-	03/23/20	211 articles
Global Health<br><br>(Ovid)<br><br>1910-	(dengue OR breakbone fever* OR break bone fever*).mp. OR exp dengue/<br><br>AND<br><br>(fortified OR micronutrient* OR supplement* OR vitamin* OR retinol OR retinoic acid* OR antioxidant* OR zinc OR iron OR thiamine OR riboflavin OR niacin OR pantothenic acid* OR pyridoxine OR biotin OR folic acid* OR folate OR cobalamin OR chromium OR magnesium).mp.<br><br>Limits: English, 2010-	03/23/20	211 articles<br><br>- 52 duplicates<br><br>= 159 articles
Scopus<br><br>1960-	(TITLE-ABS-KEY(dengue OR "breakbone fever" OR "break bone fever")) AND (TITLE-ABS-KEY (fortified OR micronutrient* OR supplement* OR vitamin* OR retinol OR retinoic acid* OR antioxidant* OR zinc OR iron OR	03/23/20	164 articles<br><br>- 34 duplicates

	thiamine OR riboflavin OR niacin OR “pantothenic acid” OR pyridoxine OR biotin OR “folic acid” OR cobalamin OR chromium OR magnesium*) AND PUBYEAR > 2009)		= 130 articles
Academic Search Complete<br><br>(Ebsco)	(dengue OR "breakbone fever" OR "break bone fever")<br><br>AND<br><br>(fortified OR micronutrient* OR supplement* OR vitamin* OR retinol OR retinoic acid* OR antioxidant* OR zinc OR iron OR thiamine OR riboflavin OR niacin OR “pantothenic acid” OR pyridoxine OR biotin OR “folic acid” OR folate OR cobalamin OR chromium OR magnesium*)<br><br>Limits: English, 2010-, Peer-reviewed	03/23/20	212 articles<br><br>- 6 duplicates<br><br>= 206 articles
Embase<br><br>1947-	‘dengue’/exp OR ‘dengue’<br><br>AND<br><br>fortified OR micronutrient* OR supplement* OR vitamin* OR retinol OR retinoic acid* OR antioxidant* OR zinc OR iron OR thiamine OR riboflavin OR niacin OR pantothenic acid* OR pyridoxine OR biotin OR folic acid* OR folate OR cobalamin OR chromium OR magnesium<br><br>Limits: English, 2010-	03/23/20	446 articles<br><br>-224 duplicates<br><br>=222 articles
		**De-duplicated in EndNote:**	**928 articles**
